# Comparing the biological response to immediate versus delayed implants placement in terms of osseointegration and bone quality

**DOI:** 10.6026/973206300221695

**Published:** 2026-03-31

**Authors:** Gurmeet Ahuja, Sapna Pandey, Shreya Gill, Mohit Mahesh Galani, Zameer Pasha, Sooriaprakas Chandrasekaran, Anukriti Kumari

**Affiliations:** 1 Department of Dentistry, Royaldente, Gurgaon, Haryana, India; 2 Department of Oral & Maxillofacial Surgery, Chandra Dental College and Hospital, Dharsania, Barabanki, Uttar Pradesh, India; 3 Department of Conservative Dentistry and Endodontics, DMD, Virginia, USA; 4 Department of Dentistry, AIIMS, Rajkot, Gujarat, India; 5 Department of Oral and Maxillofacial Surgery, Durrat Al-Alammi Dental Clinic, Al-Majmaah, Riyadh, Saudi Arabia; 6 Department of Conservative Dentistry and Endodontics, Meenakshi Ammal Dental College and Hospital, Meenakshi Academy of Higher Education and Research (Deemed to be University) Chennai, Tamil Nadu, India; 7 Department of Oral Medicine and Radiology, School of Dental Sciences, Sharda University, Greater Noida, Uttar Pradesh, India

**Keywords:** Dental implant placement, immediate implants, delayed implants, osseointegration, peri-implant bone quality

## Abstract

The timing of implant placement after tooth extraction plays a critical role in osseointegration, peri-implant healing and bone quality. Therefore, it is of interest to compare the biological responses of immediate versus delayed implant placement on implant stability and bone quality. Hence, a total of 100 participants were divided into two groups, with immediate and delayed implant placements. Thus, we show delayed implants exhibited superior early stability and bone density, but after 6 months, no significant differences were observed.

## Background:

Since the introduction of oral implants in the 1960s, their efficacy has been well-established, with no contraindications for their use in tooth replacement [1]. The success of a dental implant is largely dependent on osseointegration, the process where the implant forms a direct connection with the bone. Osseointegration is influenced by local and systemic factors such as bone quality, implant surface properties, surgical technique and the timing of implant placement [2]. Traditionally, implants are placed several months after tooth extraction, allowing the bone to heal [3]. Studies suggest that delayed placement is biologically safe, as it ensures inflammation resolution and mature bone formation. However, a dvances in implant design and surgical protocols have made immediate implant placement more common, offering benefits such as shorter treatment time, fewer surgeries and better preservation of alveolar bone [4]. Despite these benefits, immediate placement involves placing the implant in a socket with ongoing inflammation and healing tissues, which may affect early bone healing and osseointegration [5, 6]. In contrast, delayed placement allows for more mature and mineralized bone, leading to better initial stability and bone quality. However, this healing period can result in significant bone loss, which may affect implant positioning and aesthetics [7, 8]. The timing of implant placement influences bone remodelling and the quality of bone-implant contact, with immediate implants often surround by woven bone, while delayed implants are surrounded by more mature bone.

Osseointegration is a dynamic process and disturbances during the early healing phase can compromise stability [9, 10]. Improvements in implant surface topography and coatings could mitigate some of the disadvantages of immediate placement. Comparing immediate and delayed placements should go beyond survival rates to include factors like bone quality, marginal bone level changes and bone-implant contact, which can impact long-term, implant longevity and peri-implant health [11, 12]. Individual factors, such as bone density, socket design, systemic health and occlusal loading patterns, also affect the outcomes of implant placement. Although numerous studies have evaluated these protocols, conclusions on their biological behavior remain inconsistent [13, 14]. Further research focusing on osseointegration and bone quality, rather than just implant survival, is needed to provide more conclusive findings. An improved understanding of the biological responses to different timing protocols can help clinicians tailor treatments for better long-term results [15]. Therefore, it is of interest to assess the differences in osseointegration and bone quality between immediate and delayed implant placements, with the aim of improving the predictability of implant success.

## Methodology:

The current research is a prospective, randomized, controlled clinical study, which compares the biological response to immediate implant placement and delayed implant placement concerning osseointegration and peri-implant bone quality. The review and approval of the study protocol were done by the Institutional Ethics Committee and all are followed according to the Declaration of Helsinki. All participants provided written informed consent before the enrolment. For the study of 100 patients who required single-tooth extraction followed by implant replacement was included. The sample size was calculated so that there was enough power to detect a difference in osseointegration and bone quality parameters between the groups. The inclusion criteria for this study are patients aged between 18 and 55 years who require single-tooth extraction in the maxillary or mandibular posterior region. Eligible patients must have adequate bone volume to receive a standard-diameter implant without the need for extensive bone grafting, maintain good oral hygiene and have controlled periodontal health. Additionally, patients must not have any acute infection at the extraction site. The exclusion criteria include systemic conditions that affect bone healing, such as uncontrolled diabetes or osteoporosis, a history of radiotherapy or chemotherapy in the head and neck region, heavy smoking (more than 10 cigarettes/day) pregnancy or lactation and the presence of parafunctional habits like bruxism. Randomization was performed using a computer-generated random sequence. Allocation concealment was achieved using sealed opaque envelopes opened at the time of surgery. All implants used in the study had similar macro design and surface characteristics to minimize variability. Under standardized aseptic conditions, all surgical procedures were performed by one expert clinician.

Participants were randomly allocated into two equal groups:

[1] Group I (Immediate Implant Group): 50 patients receiving implant placement immediately after tooth extraction.

[2] Group II (Delayed Implant Group): 50 patients receiving implant placement after a healing period of 12 weeks following extraction.

## Immediate implant group:

Atraumatic extraction was carried out, preserving the socket walls. The implant was placed directly into the extraction socket, engaging the apical bone to achieve primary stability. Any residual gap between the implant and socket walls was left to heal naturally or filled with particulate bone graft when necessary.

## Delayed implant group:

Following atraumatic extraction, the socket was allowed to heal for 12 weeks.

[1] Implant placement was then performed using a conventional drilling protocol.

[2] Insertion torque values helped us assess the primary implant stability at placement.

[3] All patients received the same analgesics, antibiotics and in-hospital instructions.

[4] The mandible was non-loading for 3 months while the maxilla was non-loading for 4 months before loading the prosthesis.

## Outcome measures:

## Osseointegration assessment:

Implant stability was evaluated using resonance frequency analysis at placement, at 3 months and at 6 months. Clinical implant success was assessed based on absence of mobility, pain, or peri-implant infection.

## Bone quality assessment:

Peri-implant bone density was assessed using cone-beam computed tomography at baseline, 3 months and 6 months. Changes in marginal bone levels were measured radiographically using standardized reference points.

## Statistical analysis:

Data were analyzed using statistical software. Descriptive statistics were calculated for all variables. Intergroup comparisons were performed using independent t-tests or Mann-Whitney U tests, while intragroup comparisons over time were analyzed using paired t-tests or repeated measures ANOVA. A p-value of <0.05 was considered statistically significant.

## Results:

A total of 100 patients were enrolled and completed the study, with 50 patients in the immediate implant group (Group I) and 50 patients in the delayed implant group (Group II). No dropouts or implant failures were observed during the 6-month follow-up period, resulting in a 100% implant survival rate in both groups. The demographic distribution and baseline clinical parameters were comparable between the two groups, with no statistically significant differences (p > 0.05), indicating successful randomization (Table 1 - see PDF). Resonance frequency analysis showed that primary stability at placement was significantly higher in the delayed implant group (p < 0.001). However, both groups demonstrated a progressive increase in implant stability over time. At 6 months, no statistically significant difference was observed between the groups, indicating successful osseointegration (Table 2 - see PDF). Cone-beam computed tomography analysis revealed that the delayed implant group had significantly higher bone density at baseline and 3 months (p < 0.05). However, by 6 months, bone density values were comparable between the groups, suggesting active bone remodeling and maturation around immediately placed implants (Table 3 - see PDF). Both groups demonstrated minimal marginal bone loss during the follow-up period. The immediate implant group showed slightly higher bone loss at 6 months; however, the difference was not statistically significant (Table 4 - see PDF). Multivariate linear regression analysis was performed using STATA to evaluate the influence of implant placement timing on implant stability and bone density at 6 months, adjusting for age, gender and implant site. Timing of implant placement was not a significant predictor of 6-month implant stability or bone density (Table 5 - see PDF). Through delayed placement of implants showed better initial stability and bone density, immediate implants showed constant improvement over time. After 6 months of analyzing the clinical effects of a laser protocol for immediate implant placement, it can be seen that the timing did not significantly impact osseointegration or bone density.

## Discussion:

In the current research looking at osseointegration and bone quality following immediate and delayed implant placement, we saw that while delayed implants had higher primary stability and increased bone density at an earlier stage, both approaches had similar osseointegration and bone quality at 6 months. These findings indicate that, despite potential immediate biological difficulties with immediate placement, appropriate surgical technique and healing protocol can lead to comparable long-term bone remodelling and outcome to delayed placement. Our results are in accordance with several past trials and systematic reviews, there are similarities and differences with previously published literature. Schropp *et al.* (2003) [16] conducted a well-designed prospective clinical study comparing bone healing following immediate versus delayed titanium implant placement in extraction sockets. They reported slightly higher survival rates and greater crestal bone width reduction in delayed placements compared to immediate placement over 3 months, reflecting more favorable anatomical healing conditions with delayed insertion. This parallels our observation that early peri-implant bone quality tended to be higher in the delayed group. Younis *et al.* (2009) [17] evaluated bone healing and marginal bone remodeling following immediate and delayed implant placements and found that despite initial bone defect reductions in the immediate group, the magnitude of remodeling was greater in immediate implants, indicating that this protocol supports spontaneous bone healing given adequate clinical conditions. Our study likewise showed significant increases in bone density and stability over time in the immediate group, albeit starting with lower primary stability. A more recent clinical comparison by Singh *et al.* (2021) [18] reported that delayed implant placement demonstrated better crestal bone healing at 3 and 6 months when compared to immediate placement, particularly in non-grafted sockets. This supports the concept that delayed placement initially offers a more conducive environment for bone apposition, an observation consistent with our higher early bone density and marginal bone levels seen in the delayed group. In contrast, the systematic review and meta-analysis by Garcia-Sanchez *et al.* (2022) [19] concluded that implant survival rates were similar between immediate and delayed placement and although immediate placements showed higher early complications, overall outcomes, including bone level changes, did not differ significantly. These results corroborate our findings that long-term osseointegration and marginal outcomes may converge, despite initial biological differences. Another recent report by Mishra *et al.* (2024) [20] comparing crestal bone levels with and without bone grafting found greater bone loss in immediate placements compared with delayed, although graft augmentation appeared to mitigate this difference. This aligns with our observation that bone grafting or augmentation can positively influence immediate implant outcomes, reducing early bone deficiencies.

## Conclusion:

Both immediate and delayed implant placement protocols demonstrated successful osseointegration and high implant survival. Delayed implants showed better primary stability and bone density, with both methods showing similar outcomes at 6 months. When proper case selection and surgical protocols are followed, both protocols can be clinically reliable.

## Limitations:

In spite of the strengths of the present study, several limitations need to be acknowledged. A follow-up period of just 6 months does not allow assessing bone stability around the implant or the survival of the implant with loading. The clinical, radiographic and stability measures were the primary focus of this study. The study did not include any histomorphometric or micro-CT analyses. This means we did not obtain information on bone-implant contact and the microarchitecture of the bone. Even though randomization was done, the research was done at a single centre, which limits the applicability of the results to the general population and varied clinical settings. Variations in skin texture and sensation among individuals may also have affected outcomes despite standardized wound access. It is important to note that while clinically relevant, the use of CBCT-derived bone density values may not represent true bone mineral density. Lastly, although the differences in implant position and the occlusal forces of patients could not be completely controlled, they may have affected early stability and the bone remodeling pattern.
